# Supplementary material for: Genetic association of inflammatory marker GlycA with lung function and respiratory diseases
Source: Nat Commun. 2024 May 4;15:3751. doi: 10.1038/s41467-024-47845-w (PMC11069551; doi:10.1038/s41467-024-47845-w)
Supplement: Supplementary file 1 — Supplementary Information [file 41467_2024_47845_MOESM1_ESM.pdf]

Supplementary Information

Genetic association of novel inflammatory marker GlycA with lung function and respiratory diseases

Yanjun Guo [1,2,3,*], Quanhong Liu[1,2], Zhilin Zheng[1,2], Mengxia Qing[1,2], Tianci Yao [4], Bin Wang[1,2], Min Zhou[1,2], Dongming Wang[1,2], Qinmei Ke [4], Jixuan Ma[1,2], Zhilei Shan[5], Weihong Chen[1,2,*]

[1] Department of Occupational and Environmental Health, School of Public Health, Tongji Medical College, Huazhong University of Science and Technology, Wuhan, China

[2] Key Laboratory of Environment and Health, Ministry of Education & Ministry of Environmental Protection, School of Public Health, Tongji Medical College, Huazhong University of Science and Technology, Wuhan, Hubei 430030, China

[3] Program in Genetic Epidemiology and Statistical Genetics, Department of Epidemiology, Harvard T.H. Chan School of Public Health, Boston, MA, 02215, USA

[4] Department of Geriatrics, Union Hospital, Tongji Medical College, Huazhong University of Science and Technology, Wuhan, China

[5] Department of Nutrition and Food Hygiene, School of Public Health, Tongji Medical College, Huazhong University of Science and Technology, Wuhan, China

***Corresponding author:**

Dr. Yanjun Guo
Department of Occupational and Environmental Health, School of Public Health, Tongji Medical College, Huazhong University of Science and Technology, Wuhan, Hubei 430030, China.
E-mail: yanjunguo@hsph.harvard.edu

Dr. Weihong Chen
Department of Occupational and Environmental Health, School of Public Health, Tongji Medical College, Huazhong University of Science and Technology, Wuhan, Hubei 430030, China.
E-mail: wchen@tjmu.edu.cn

Contents

Supplementary Figure 1 Partitioned genetic correlations of WBC with lung function parameters (FEV1, FVC, FEV1/FVC ratio, and PEF), asthma, and COPD according to 11 functional categories using linkage disequilibrium score regression (LDSC). 3
Supplementary Figure 2 Partitioned genetic correlations of hsCRP with lung function parameters (FEV1, FVC, FEV1/FVC ratio, and PEF), asthma, and COPD according to 11 functional categories using linkage disequilibrium score regression (LDSC). 4
Supplementary Figure 3 Partitioned genetic correlations of fibrinogen with lung function parameters (FEV1, FVC, FEV1/FVC ratio, and PEF), asthma, and COPD according to 11 functional categories using linkage disequilibrium score regression (LDSC). 5
Supplementary Figure 4 Partitioned genetic correlations of albumin with lung function parameters (FEV1, FVC, FEV1/FVC ratio, and PEF), asthma, and COPD according to 11 functional categories using linkage disequilibrium score regression (LDSC). 6
Supplementary Figure 5 GARFIELD enrichment wheel plots in DNase I–hypersensitive sites (hotspots) for shared signals between FEV1 and hsCRP 7
Supplementary Figure 6 GARFIELD enrichment wheel plots in DNase I–hypersensitive sites (hotspots) for shared signals between FVC and hsCRP 8
Supplementary Figure 7 GARFIELD enrichment wheel plots in DNase I–hypersensitive sites (hotspots) for shared signals between FEV1/FVC ratio and hsCRP 9
Supplementary Figure 8 GARFIELD enrichment wheel plots in DNase I–hypersensitive sites (hotspots) for shared signals between PEF and hsCRP 10
Supplementary Figure 9 GARFIELD enrichment wheel plots in DNase I–hypersensitive sites (hotspots) for shared signals between asthma and hsCRP 11
Supplementary Figure 10 GARFIELD enrichment wheel plots in DNase I–hypersensitive sites (hotspots) for shared signals between COPD and hsCRP 12
Supplementary Figure 11 GARFIELD enrichment wheel plots in DNase I–hypersensitive sites (hotspots) for shared signals between FEV1 and albumin 13
Supplementary Figure 12 GARFIELD enrichment wheel plots in DNase I–hypersensitive sites (hotspots) for shared signals between FVC and albumin 14
Supplementary Figure 13 GARFIELD enrichment wheel plots in DNase I–hypersensitive sites (hotspots) for shared signals between FEV1/FVC ratio and albumin 15
Supplementary Figure 14 GARFIELD enrichment wheel plots in DNase I–hypersensitive sites (hotspots) for shared signals between PEF and albumin 16
Supplementary Figure 15 GARFIELD enrichment wheel plots in DNase I–hypersensitive sites (hotspots) for shared signals between Asthma and albumin 17
Supplementary Figure 16 GARFIELD enrichment wheel plots in DNase I–hypersensitive sites (hotspots) for shared signals between COPD and albumin 18

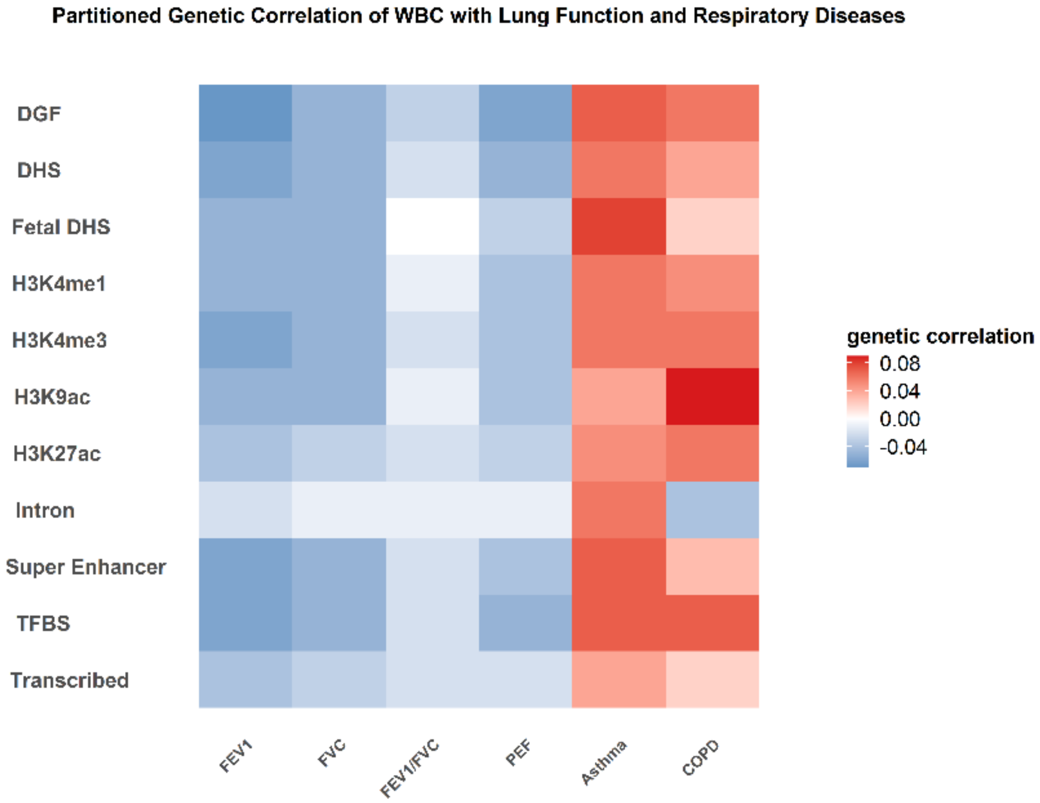

Supplementary Figure 1 **Partitioned genetic correlations of WBC with lung function parameters (FEV1, FVC, FEV1/FVC ratio, and PEF), asthma, and COPD according to 11 functional categories using linkage disequilibrium score regression (LDSC).** Colors represent the magnitude of genetic correlation of WBC with lung function parameters (FEV1, FVC, FEV1/FVC ratio, and PEF), asthma, and COPD at each functional category using LDSC, red for positive genetic correlation and blue for negative genetic correlation. Numbers represent the genetic correlation at nominal significance level (P<0.05); * represent significant genetic correlation after controlling for multiple testing (P<0.05/ [30 ×11]). Abbreviations: WBC: white blood cell; FEV1: forced expired volume in 1 second; FVC: forced vital capacity; PEF: peak expiratory flow; COPD: chronic obstructive pulmonary diseases. All P values are two-sided.

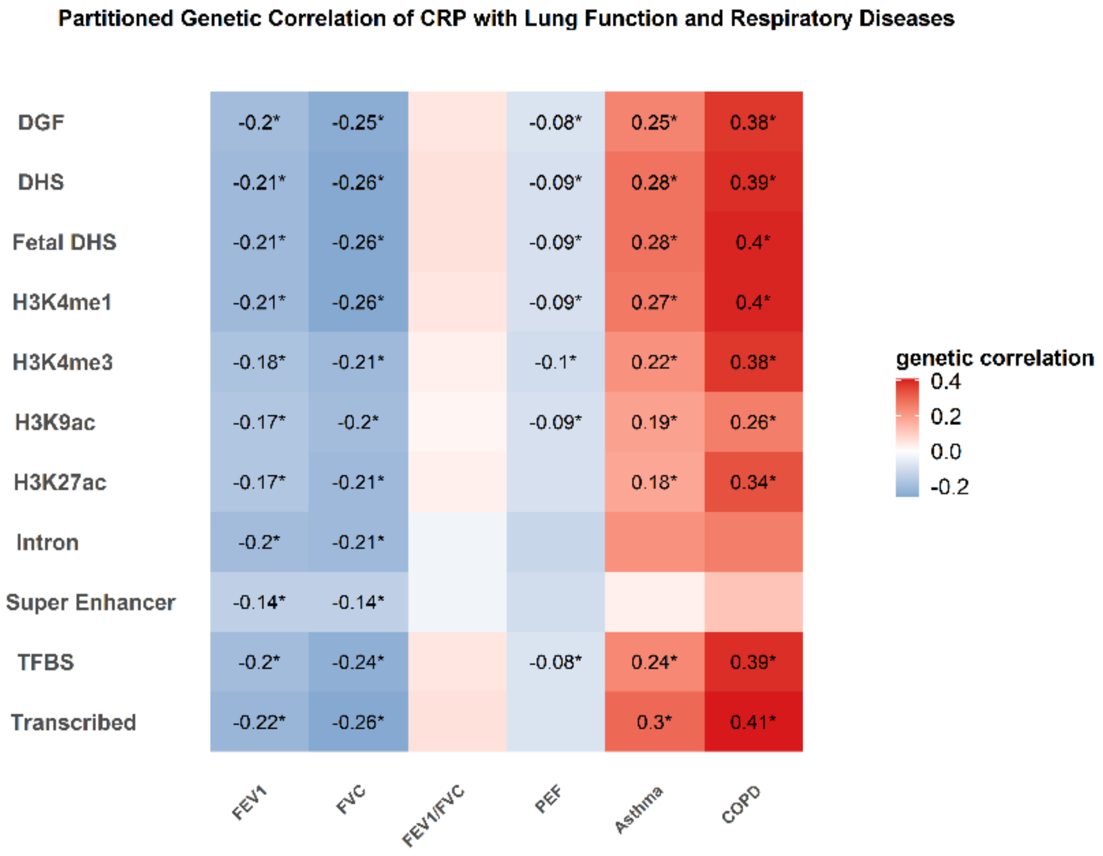

Supplementary Figure 2 Partitioned genetic correlations of hsCRP with lung function parameters (FEV1, FVC, FEV1/FVC ratio, and PEF), asthma, and COPD according to 11 functional categories using linkage disequilibrium score regression (LDSC). Colors represent the magnitude of genetic correlation of hsCRP with lung function parameters (FEV1, FVC, FEV1/FVC ratio, and PEF), asthma, and COPD at each functional category using LDSC, red for positive genetic correlation and blue for negative genetic correlation. Numbers represent the genetic correlation at nominal significance level (P<0.05); * represent significant genetic correlation after controlling for multiple testing (P<0.05/[30 ×11]). Abbreviations: hsCRP: high sensitivity C-reactive protein; FEV1: forced expired volume in 1 second; FVC: forced vital capacity; PEF: peak expiratory flow; COPD: chronic obstructive pulmonary diseases. All P values are two-sided.

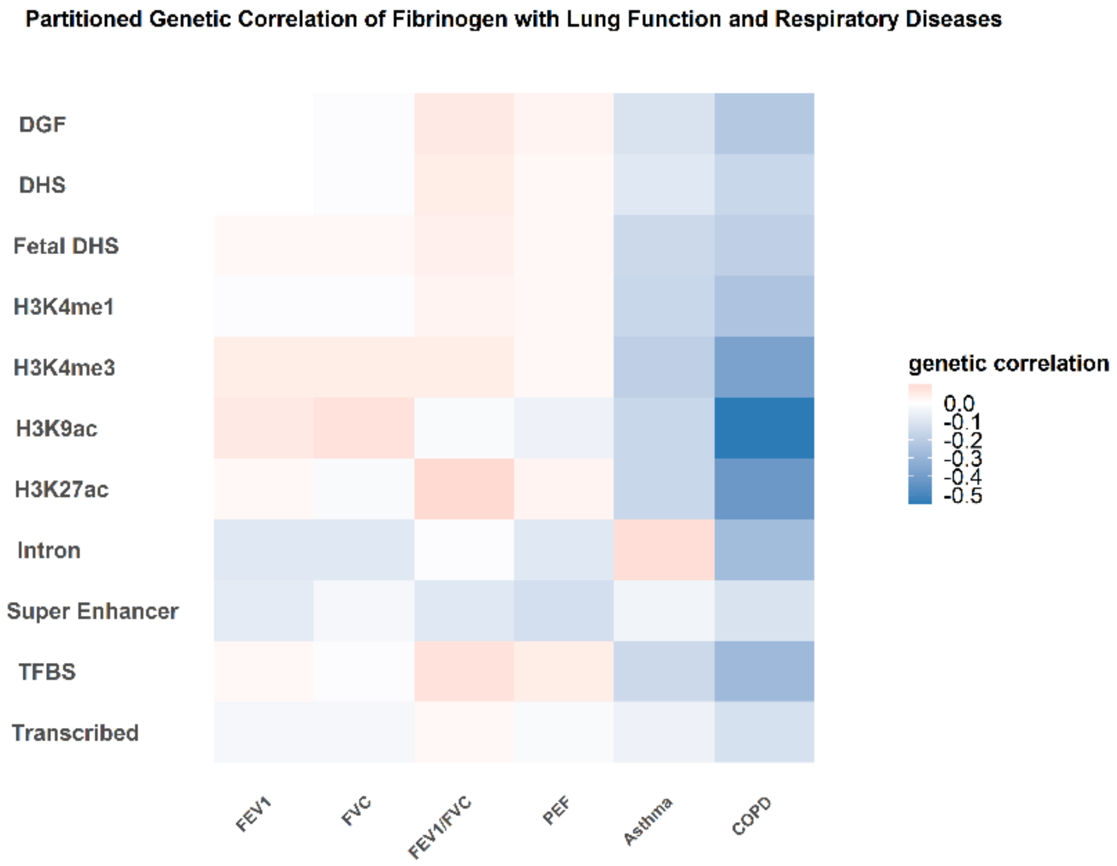

Supplementary Figure 3 **Partitioned genetic correlations of fibrinogen with lung function parameters (FEV1, FVC, FEV1/FVC ratio, and PEF), asthma, and COPD according to 11 functional categories using linkage disequilibrium score regression (LDSC).** Colors represent the magnitude of genetic correlation of fibrinogen with lung function parameters (FEV1, FVC, FEV1/FVC ratio, and PEF), asthma, and COPD at each functional category using LDSC, red for positive genetic correlation and blue for negative genetic correlation. Numbers represent the genetic correlation at nominal significance level (P<0.05); * represent significant genetic correlation after controlling for multiple testing (P<0.05/ [30 ×11]). Abbreviations: FEV1: forced expired volume in 1 second; FVC: forced vital capacity; PEF: peak expiratory flow; COPD: chronic obstructive pulmonary diseases. All P values are two-sided.

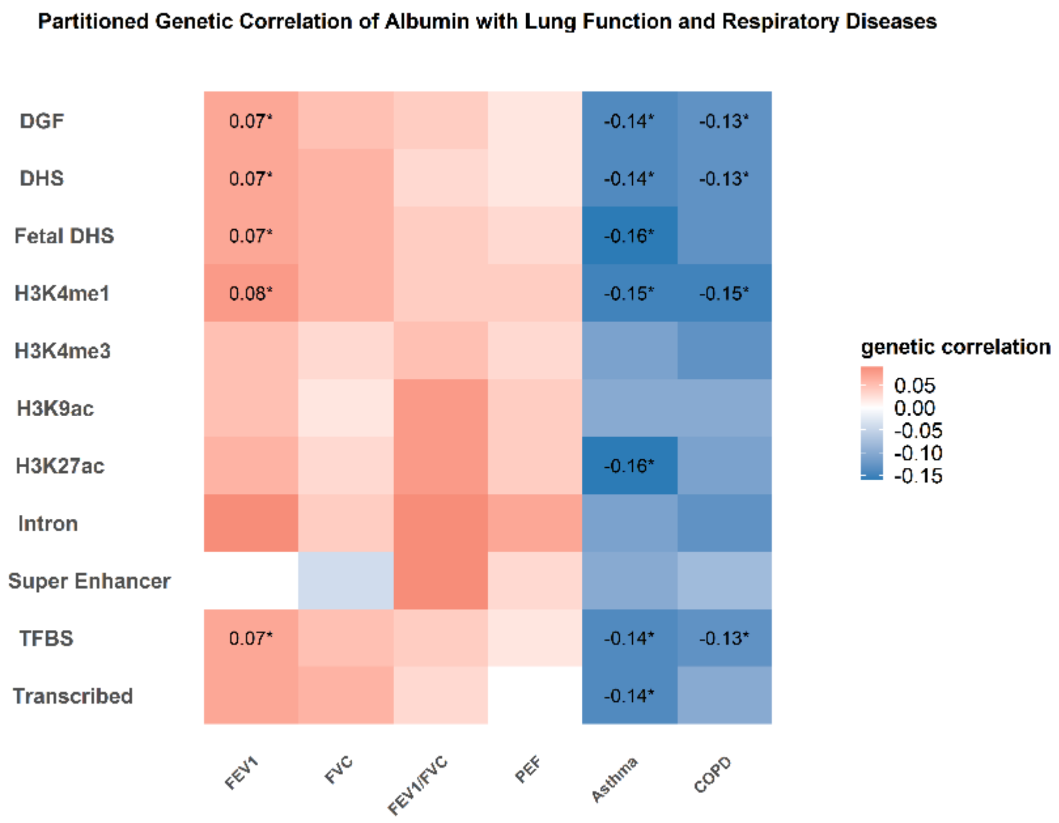

Supplementary Figure 4 **Partitioned genetic correlations of albumin with lung function parameters (FEV1, FVC, FEV1/FVC ratio, and PEF), asthma, and COPD according to 11 functional categories using linkage disequilibrium score regression (LDSC).** Colors represent the magnitude of genetic correlation of albumin with lung function parameters (FEV1, FVC, FEV1/FVC ratio, and PEF), asthma, and COPD at each functional category using LDSC, red for positive genetic correlation and blue for negative genetic correlation. Numbers represent the genetic correlation at nominal significance level (P<0.05); * represent significant genetic correlation after controlling for multiple testing (P<0.05/ [30 ×11]). Abbreviations: FEV1: forced expired volume in 1 second; FVC: forced vital capacity; PEF: peak expiratory flow; COPD: chronic obstructive pulmonary diseases. All P values are two-sided.

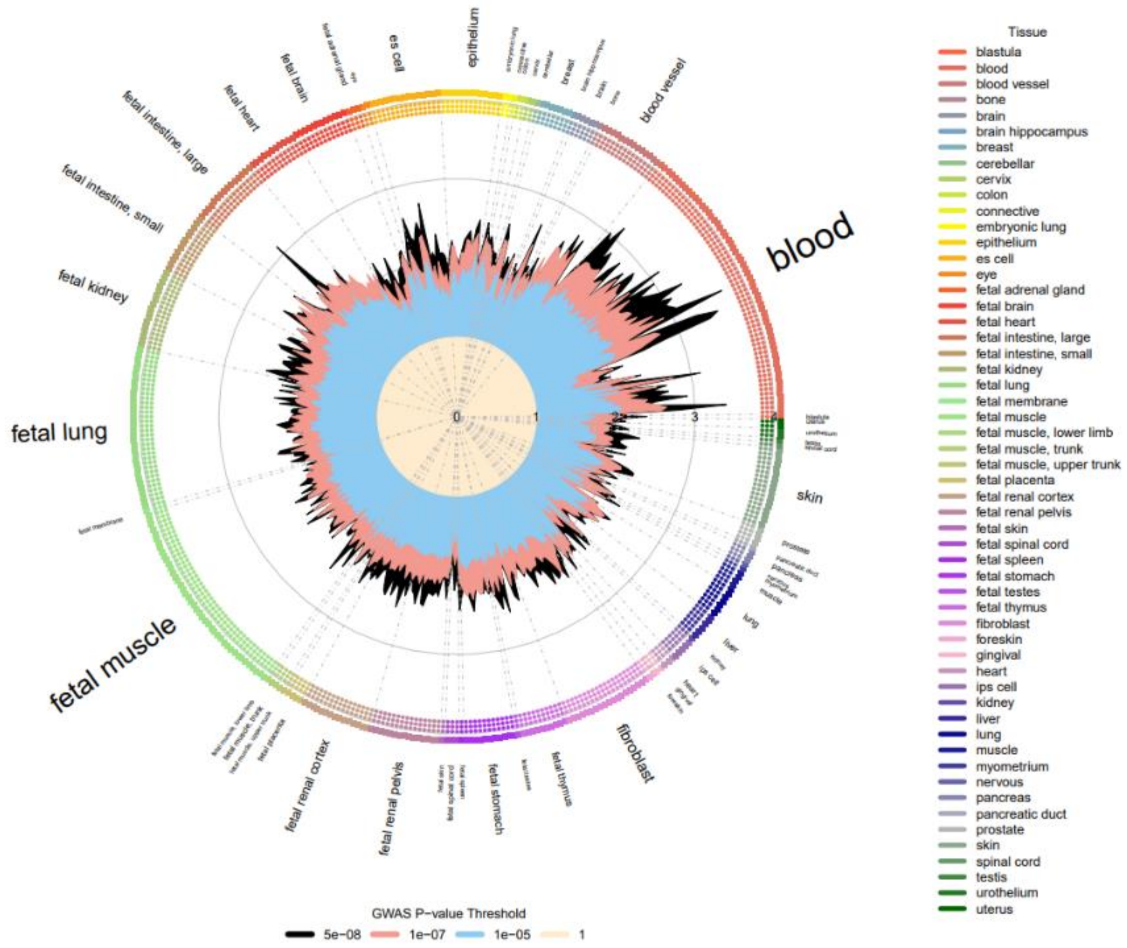

Supplementary Figure 5 GARFIELD enrichment wheel plots in DNase I–hypersensitive sites (hotspots) for shared signals between FEV1 and hsCRP. Radial lines show OR values at eight GWAS P-value thresholds (T) for all ENCODE and Roadmap Epigenomics DHS cell lines, sorted by tissue on the outer circle. Dots in the inner ring of the outer circle denote significant GARFIELD enrichment (if present) at $T < 10^{-5}$ (outermost) to $T < 10^{-8}$ (innermost) after multiple-testing correction for the number of effective annotations and are colored with respect to the tissue cell type tested (font size of tissue labels reflect the number of cell types from that tissue).

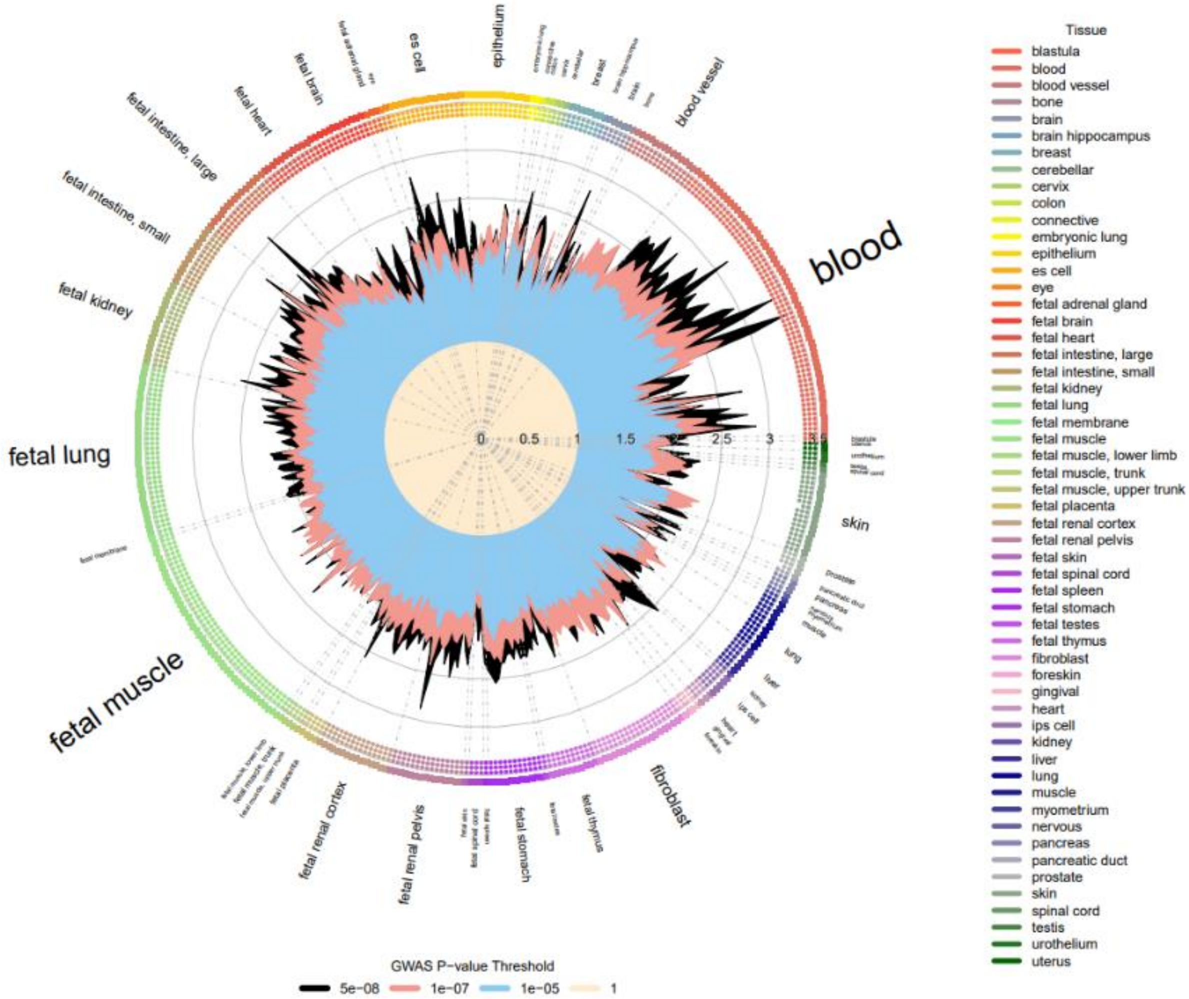

Supplementary Figure 6 **GARFIELD enrichment wheel plots in DNase I–hypersensitive sites (hotspots) for shared signals between FVC and hsCRP.** Radial lines show OR values at eight GWAS P-value thresholds (T) for all ENCODE and Roadmap Epigenomics DHS cell lines, sorted by tissue on the outer circle. Dots in the inner ring of the outer circle denote significant GARFIELD enrichment (if present) at $T < 10^{-5}$ (outermost) to $T < 10^{-8}$ (innermost) after multiple-testing correction for the number of effective annotations and are colored with respect to the tissue cell type tested (font size of tissue labels reflect the number of cell types from that tissue).

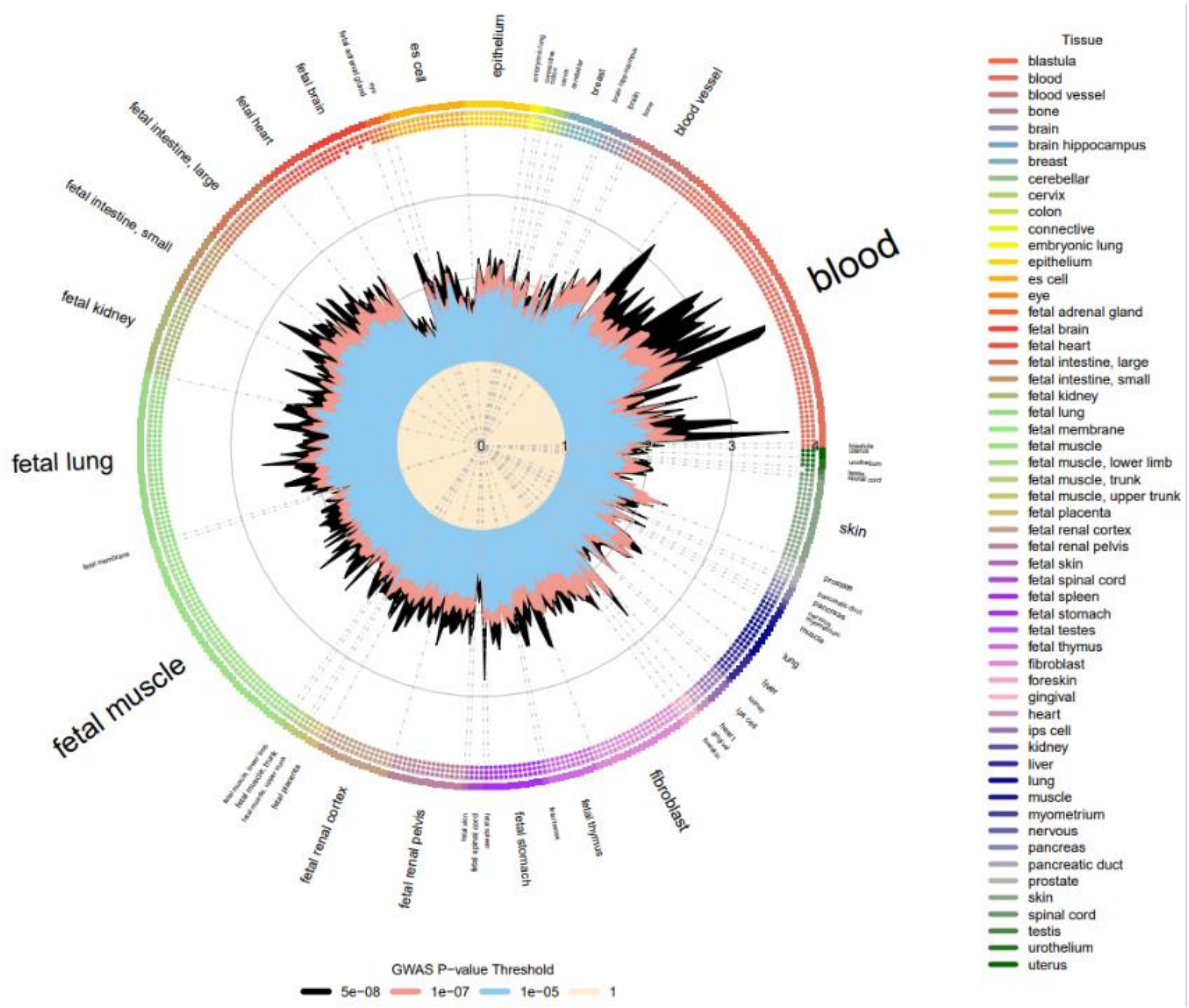

Supplementary Figure 7 **GARFIELD enrichment wheel plots in DNase I–hypersensitive sites (hotspots) for shared signals between FEV1/FVC ratio and hsCRP.** Radial lines show OR values at eight GWAS P-value thresholds (T) for all ENCODE and Roadmap Epigenomics DHS cell lines, sorted by tissue on the outer circle. Dots in the inner ring of the outer circle denote significant GARFIELD enrichment (if present) at $T < 10^{-5}$ (outermost) to $T < 10^{-8}$ (innermost) after multiple-testing correction for the number of effective annotations and are colored with respect to the tissue cell type tested (font size of tissue labels reflect the number of cell types from that tissue).

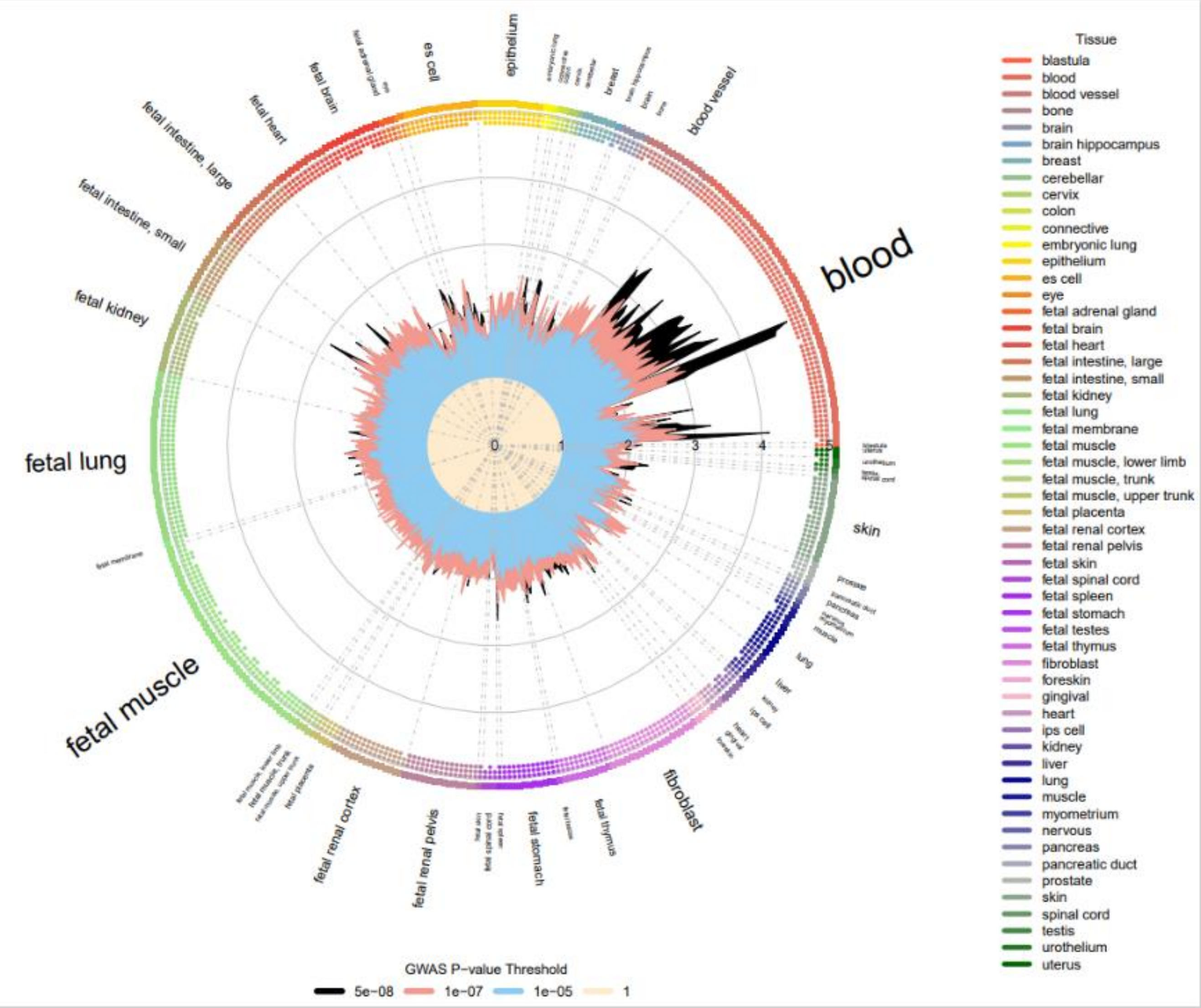

Supplementary Figure 8 **GARFIELD enrichment wheel plots in DNase I–hypersensitive sites (hotspots) for shared signals between PEF and hsCRP.** Radial lines show OR values at eight GWAS P-value thresholds (T) for all ENCODE and Roadmap Epigenomics DHS cell lines, sorted by tissue on the outer circle. Dots in the inner ring of the outer circle denote significant GARFIELD enrichment (if present) at $T < 10^{-5}$ (outermost) to $T < 10^{-8}$ (innermost) after multiple-testing correction for the number of effective annotations and are colored with respect to the tissue cell type tested (font size of tissue labels reflect the number of cell types from that tissue).

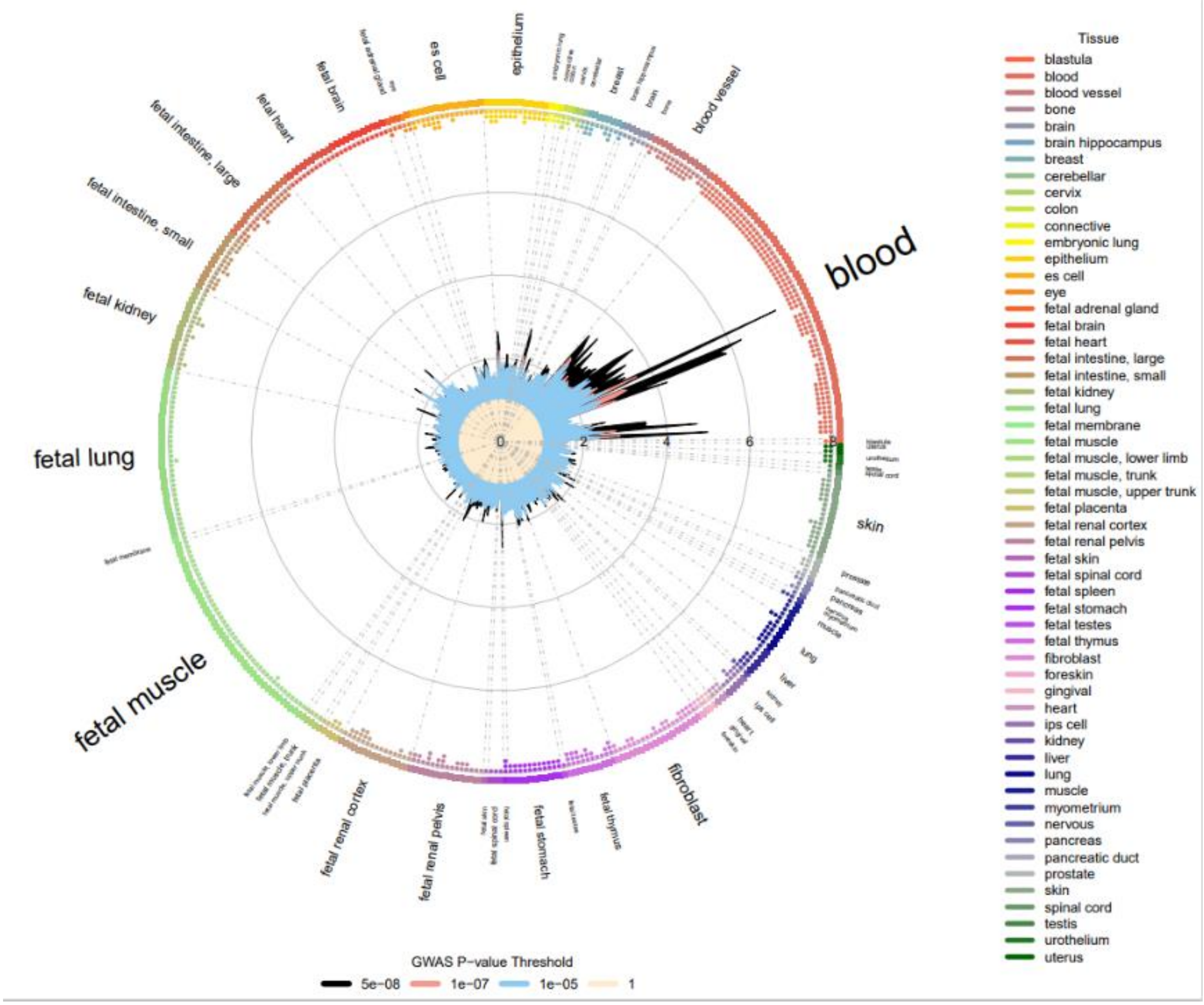

Supplementary Figure 9 **GARFIELD enrichment wheel plots in DNase I–hypersensitive sites (hotspots) for shared signals between asthma and hsCRP.** Radial lines show OR values at eight GWAS P-value thresholds (T) for all ENCODE and Roadmap Epigenomics DHS cell lines, sorted by tissue on the outer circle. Dots in the inner ring of the outer circle denote significant GARFIELD enrichment (if present) at $T < 10^{-5}$ (outermost) to $T < 10^{-8}$ (innermost) after multiple-testing correction for the number of effective annotations and are colored with respect to the tissue cell type tested (font size of tissue labels reflect the number of cell types from that tissue).

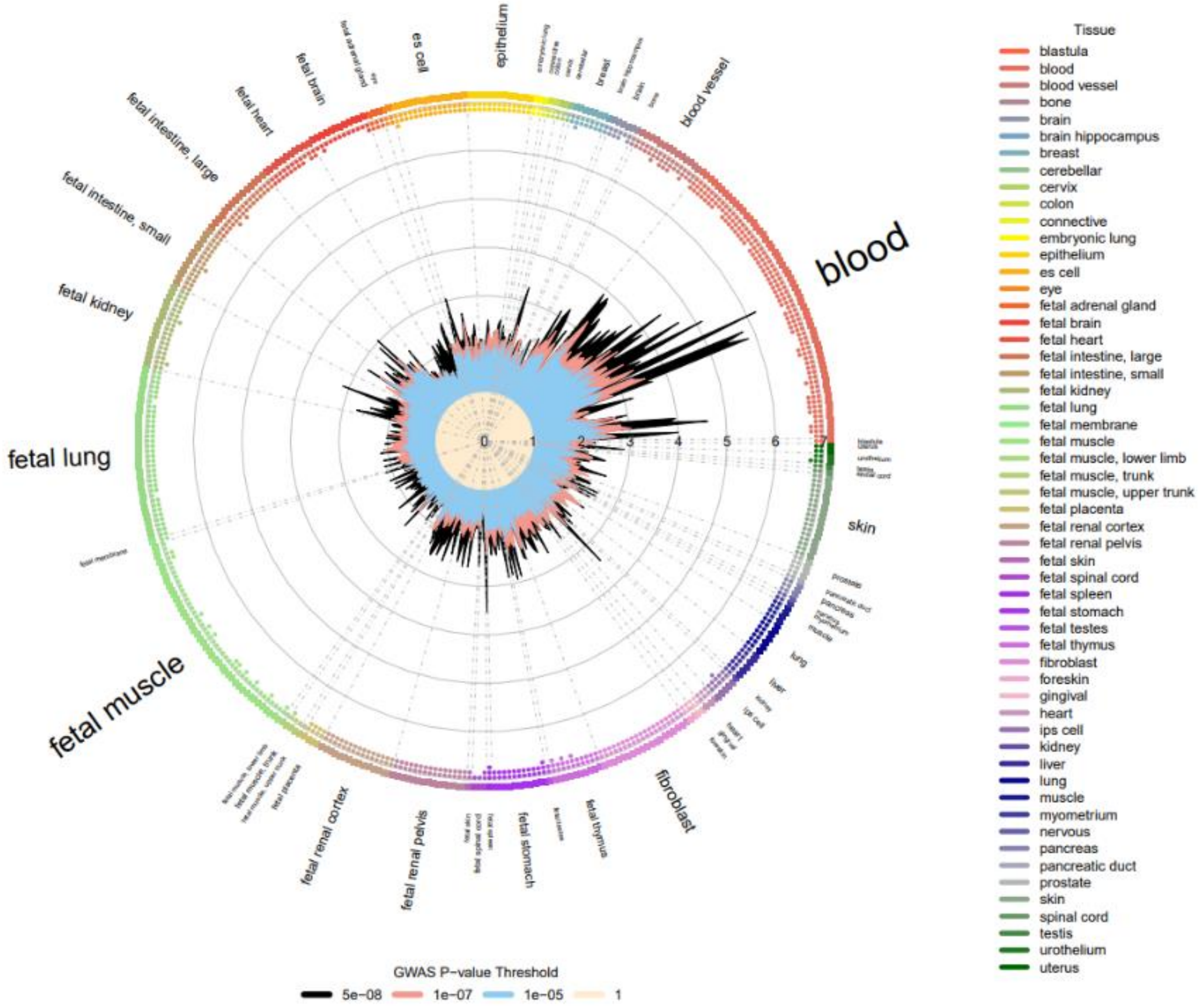

Supplementary Figure 10 **GARFIELD enrichment wheel plots in DNase I–hypersensitive sites (hotspots) for shared signals between COPD and hsCRP.** Radial lines show OR values at eight GWAS P-value thresholds (T) for all ENCODE and Roadmap Epigenomics DHS cell lines, sorted by tissue on the outer circle. Dots in the inner ring of the outer circle denote significant GARFIELD enrichment (if present) at $T < 10^{-5}$ (outermost) to $T < 10^{-8}$ (innermost) after multiple-testing correction for the number of effective annotations and are colored with respect to the tissue cell type tested (font size of tissue labels reflect the number of cell types from that tissue).

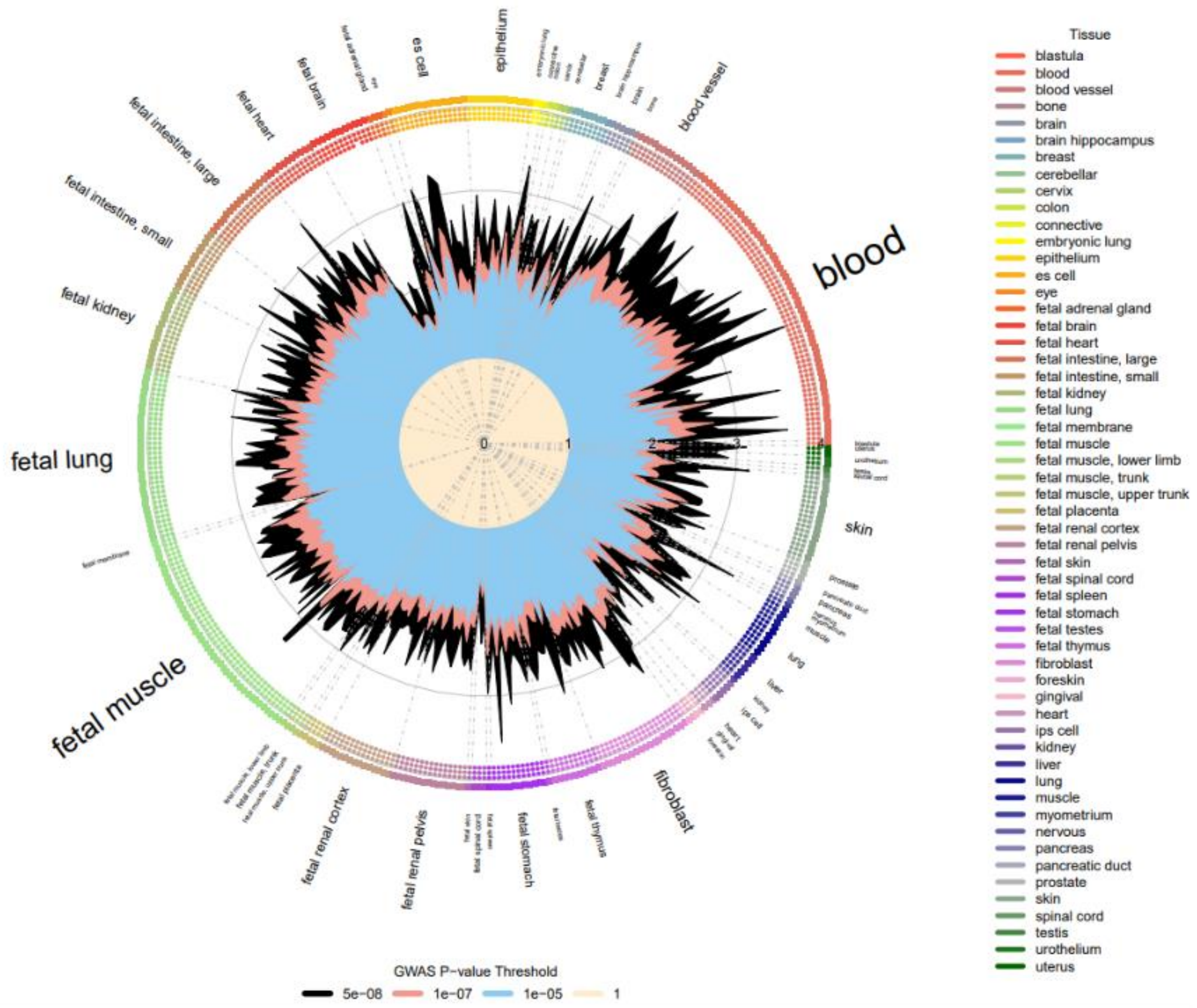

Supplementary Figure 11 **GARFIELD enrichment wheel plots in DNase I–hypersensitive sites (hotspots) for shared signals between FEV1 and albumin.** Radial lines show OR values at eight GWAS P-value thresholds (T) for all ENCODE and Roadmap Epigenomics DHS cell lines, sorted by tissue on the outer circle. Dots in the inner ring of the outer circle denote significant GARFIELD enrichment (if present) at $T < 10^{-5}$ (outermost) to $T < 10^{-8}$ (innermost) after multiple-testing correction for the number of effective annotations and are colored with respect to the tissue cell type tested (font size of tissue labels reflect the number of cell types from that tissue).

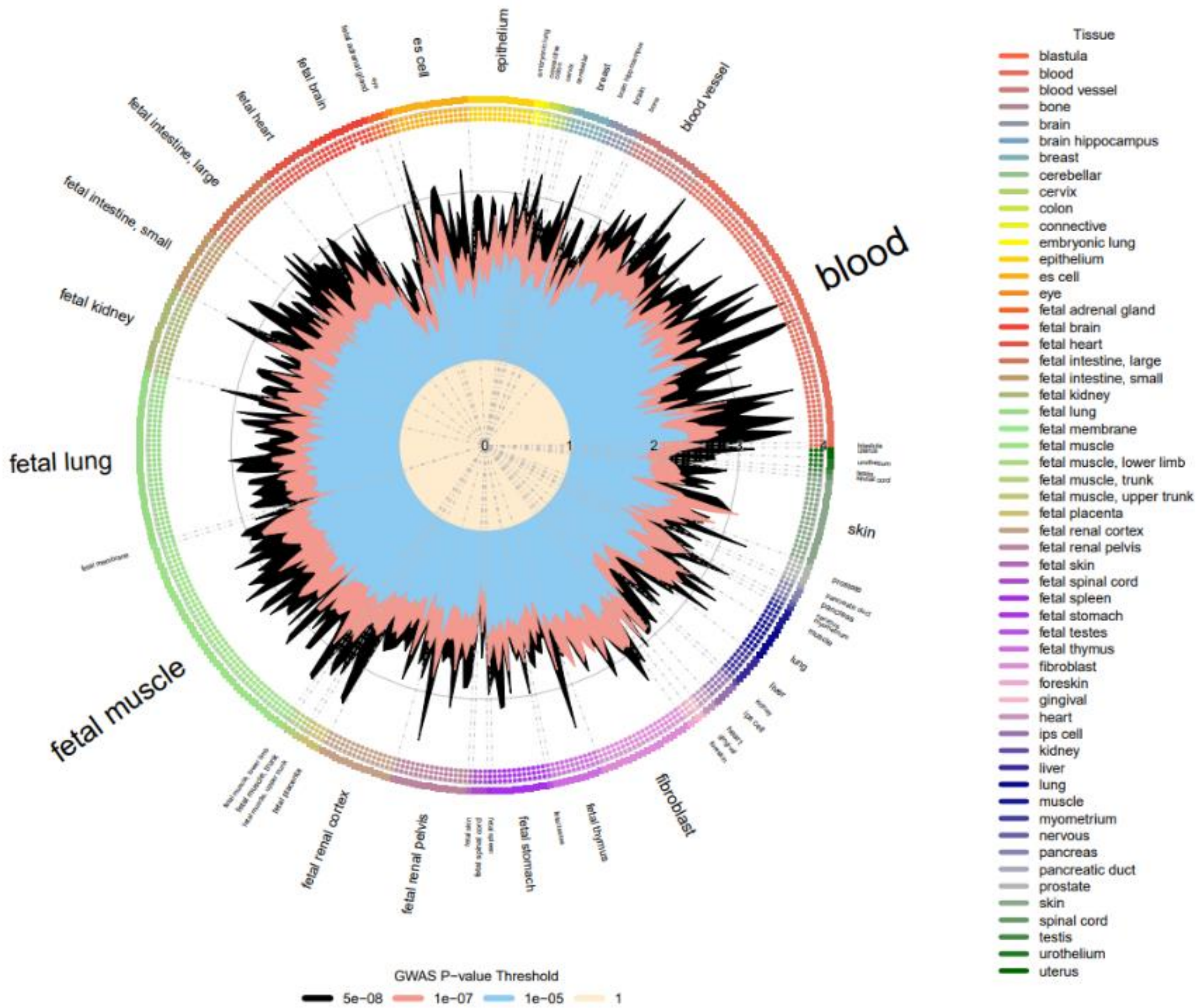

Supplementary Figure 12 **GARFIELD enrichment wheel plots in DNase I–hypersensitive sites (hotspots) for shared signals between FVC and albumin.** Radial lines show OR values at eight GWAS P-value thresholds (T) for all ENCODE and Roadmap Epigenomics DHS cell lines, sorted by tissue on the outer circle. Dots in the inner ring of the outer circle denote significant GARFIELD enrichment (if present) at $T < 10^{-5}$ (outermost) to $T < 10^{-8}$ (innermost) after multiple-testing correction for the number of effective annotations and are colored with respect to the tissue cell type tested (font size of tissue labels reflect the number of cell types from that tissue).

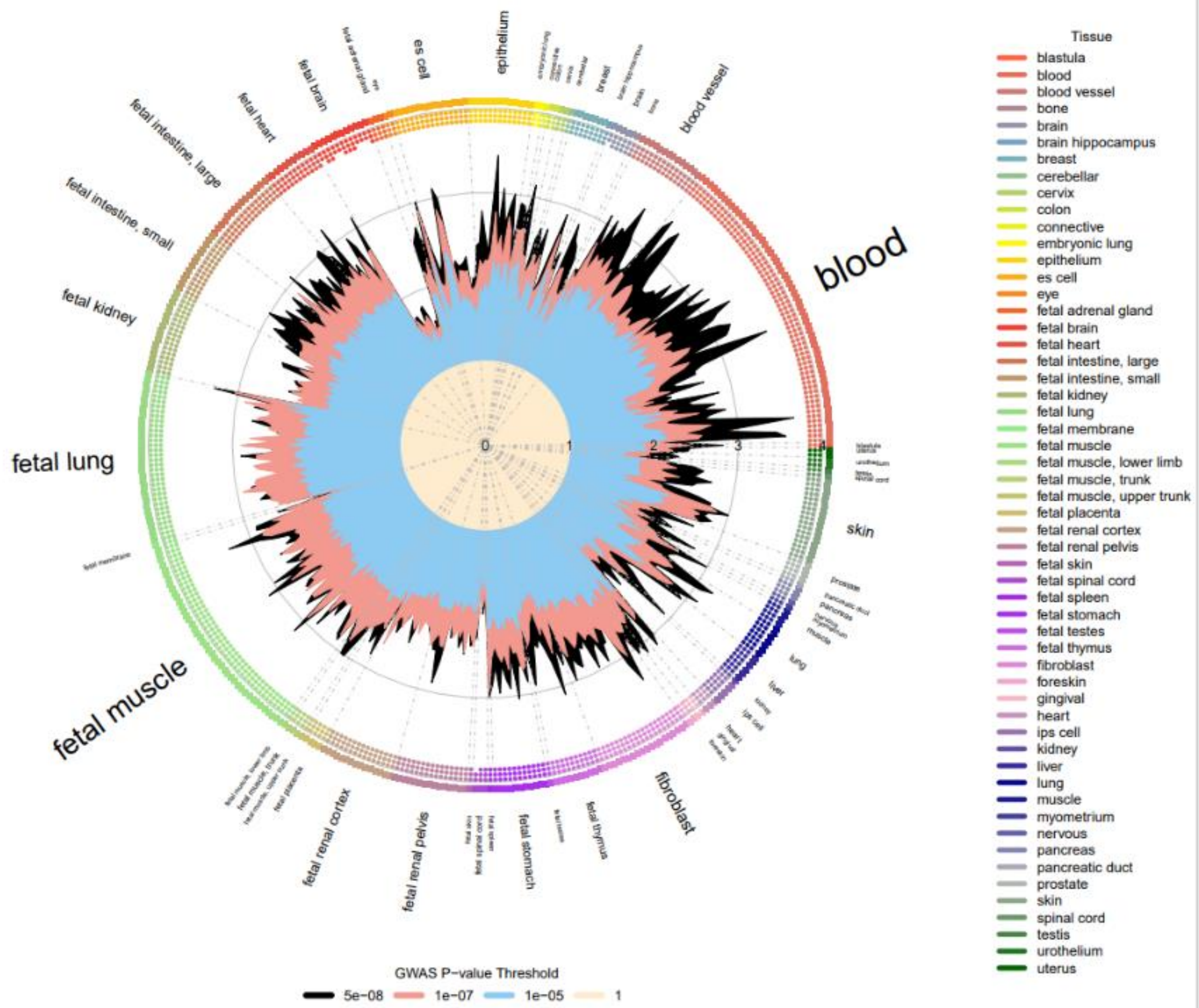

Supplementary Figure 13 **GARFIELD enrichment wheel plots in DNase I–hypersensitive sites (hotspots) for shared signals between FEV1/FVC ratio and albumin.** Radial lines show OR values at eight GWAS P-value thresholds (T) for all ENCODE and Roadmap Epigenomics DHS cell lines, sorted by tissue on the outer circle. Dots in the inner ring of the outer circle denote significant GARFIELD enrichment (if present) at $T < 10^{-5}$ (outermost) to $T < 10^{-8}$ (innermost) after multiple-testing correction for the number of effective annotations and are colored with respect to the tissue cell type tested (font size of tissue labels reflect the number of cell types from that tissue).

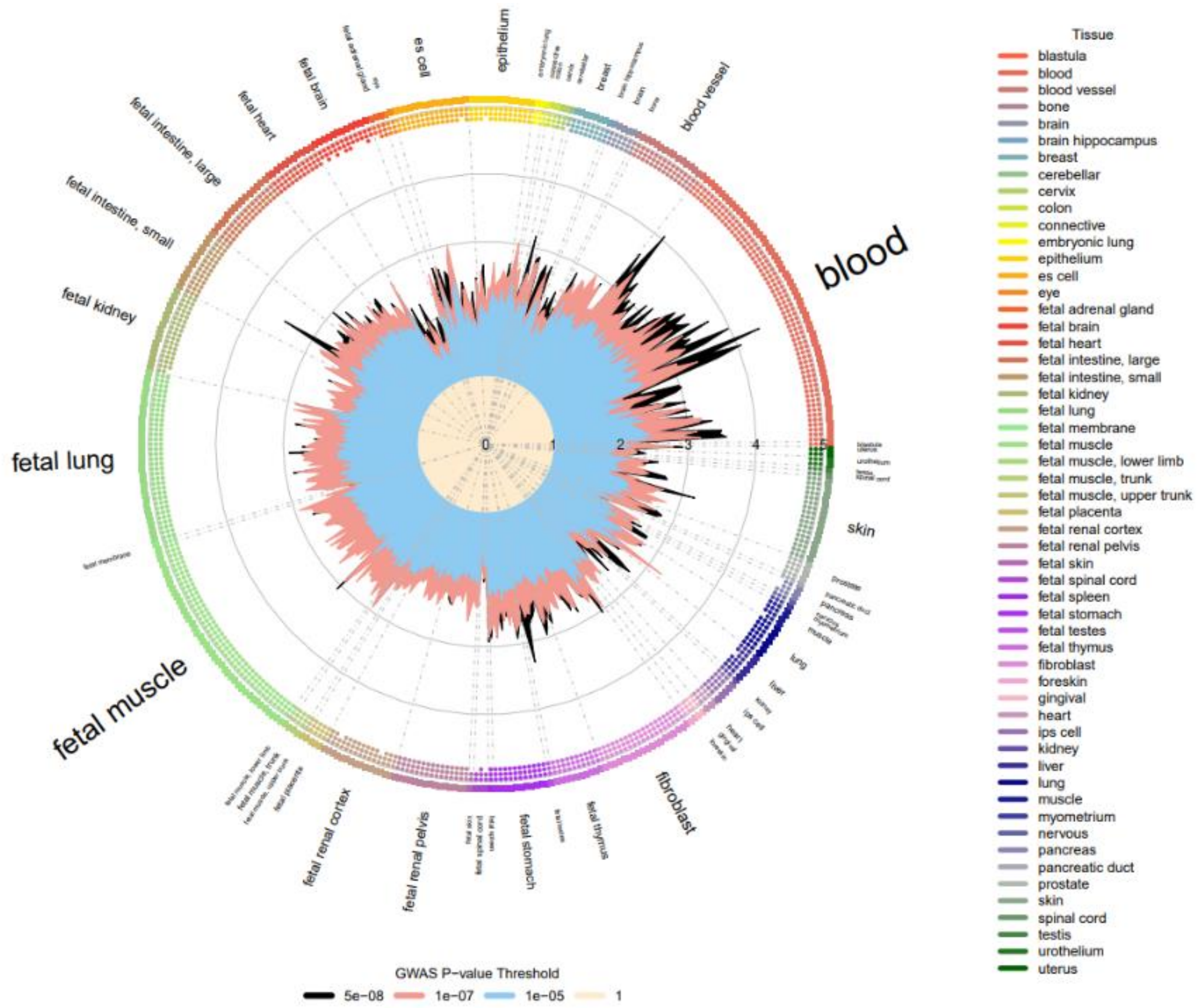

Supplementary Figure 14 **GARFIELD enrichment wheel plots in DNase I–hypersensitive sites (hotspots) for shared signals between PEF and albumin.** Radial lines show OR values at eight GWAS P-value thresholds (T) for all ENCODE and Roadmap Epigenomics DHS cell lines, sorted by tissue on the outer circle. Dots in the inner ring of the outer circle denote significant GARFIELD enrichment (if present) at $T < 10^{-5}$ (outermost) to $T < 10^{-8}$ (innermost) after multiple-testing correction for the number of effective annotations and are colored with respect to the tissue cell type tested (font size of tissue labels reflect the number of cell types from that tissue).

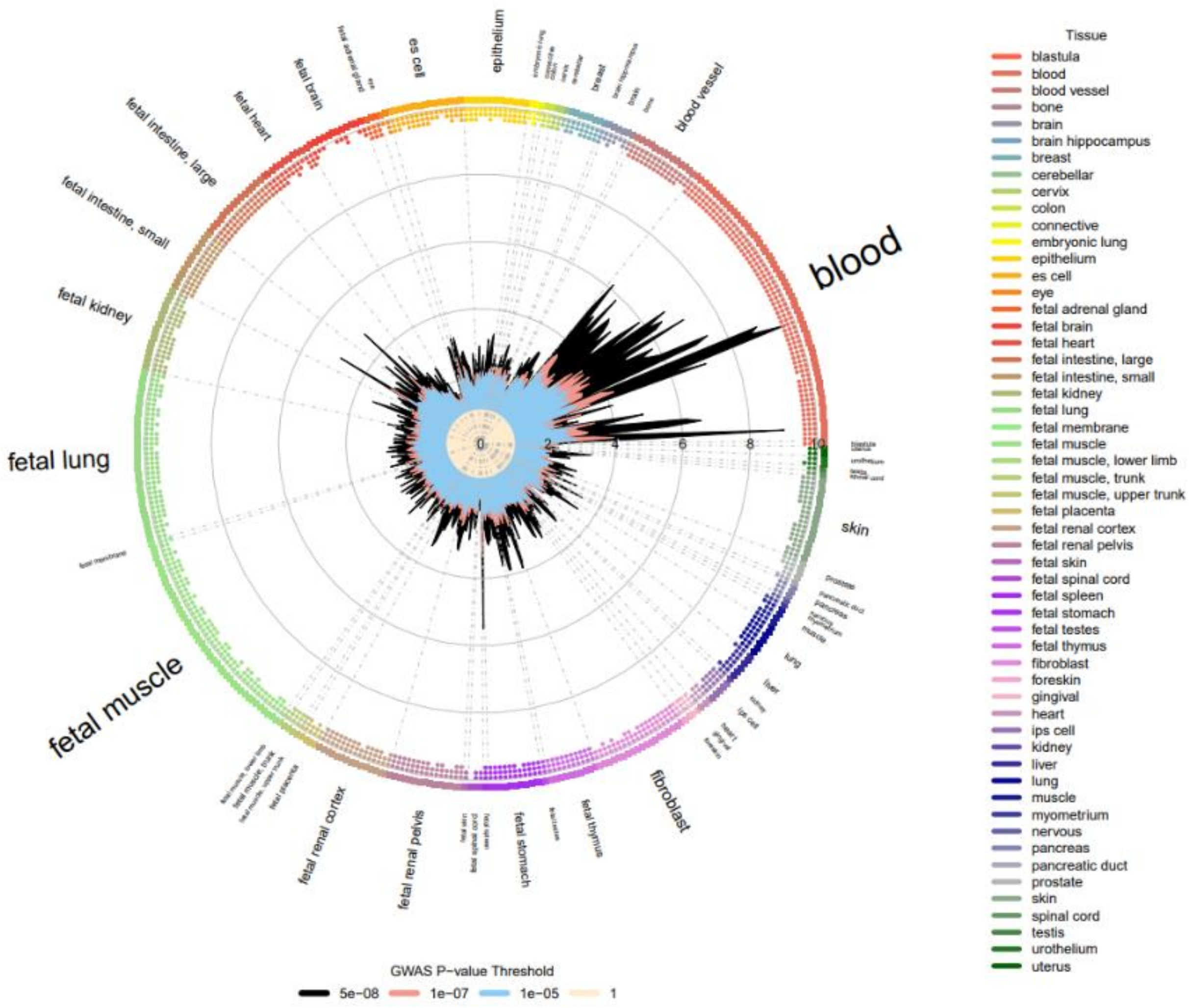

Supplementary Figure 15 **GARFIELD enrichment wheel plots in DNase I–hypersensitive sites (hotspots) for shared signals between asthma and albumin.** Radial lines show OR values at eight GWAS P-value thresholds (T) for all ENCODE and Roadmap Epigenomics DHS cell lines, sorted by tissue on the outer circle. Dots in the inner ring of the outer circle denote significant GARFIELD enrichment (if present) at T < 10^{-5} (outermost) to T < 10^{-8} (innermost) after multiple-testing correction for the number of effective annotations and are colored with respect to the tissue cell type tested (font size of tissue labels reflect the number of cell types from that tissue).

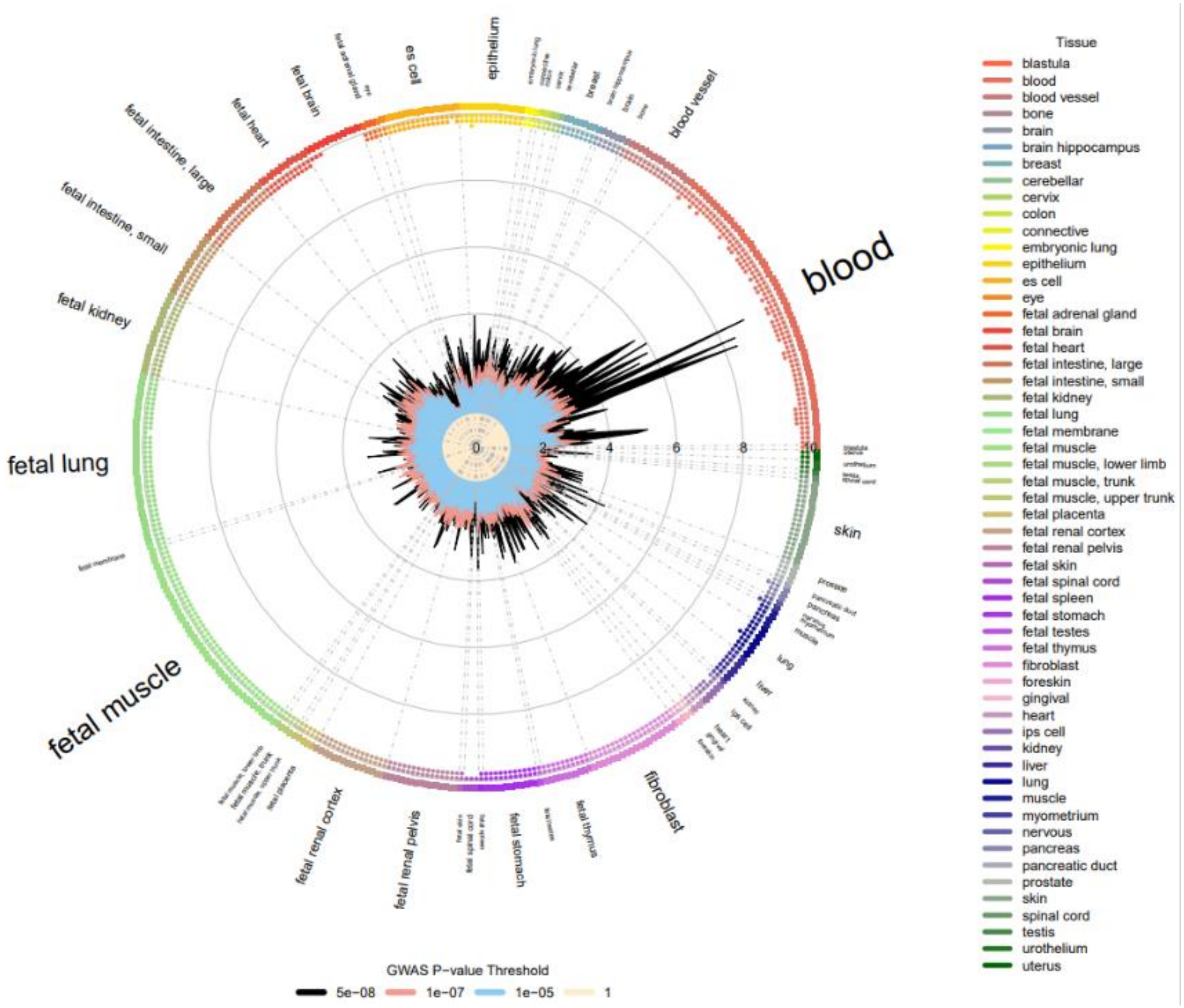

Supplementary Figure 16 **GARFIELD enrichment wheel plots in DNase I–hypersensitive sites (hotspots) for shared signals between COPD and albumin.** Radial lines show OR values at eight GWAS P-value thresholds (T) for all ENCODE and Roadmap Epigenomics DHS cell lines, sorted by tissue on the outer circle. Dots in the inner ring of the outer circle denote significant GARFIELD enrichment (if present) at $T < 10^{-5}$ (outermost) to $T < 10^{-8}$ (innermost) after multiple-testing correction for the number of effective annotations and are colored with respect to the tissue cell type tested (font size of tissue labels reflect the number of cell types from that tissue).
