# Supplementary material for: Genetic association of inflammatory marker GlycA with lung function and respiratory diseases
Source: Nat Commun. 2024 May 4;15:3751. doi: 10.1038/s41467-024-47845-w (PMC11069551; doi:10.1038/s41467-024-47845-w)
Supplement: Supplementary file 3 — Description of Additional Supplementary Files [file 41467_2024_47845_MOESM3_ESM.pdf]

Description of Additional Supplementary Files

File Name: Supplementary Data 1
Description: Information of Summary Statistics Used

File Name: Supplementary Data 2
Description: Supplementary Data 2 Sensitivity analysis of genetic correlations of glycA and other inflammatory biomarkers with lung function parameters (FEV1, FVC, FEV1/FVC ratio, and PEF) using LDSC

File Name: Supplementary Data 3
Description: Shared genetics from cross-trait meta-analysis of GlycA with lung function parameters, asthma and COPD using CPASSOC

File Name: Supplementary Data 4
Description: Shared genetics from cross-trait meta-analysis of CRP with lung function parameters, asthma and COPD using CPASSOC

File Name: Supplementary Data 5
Description: Shared genetics from cross-trait meta-analysis of albumin with lung function parameters, asthma and COPD using CPASSOC

File Name: Supplementary Data 6
Description: Shared Significant Expression-Trait Associations between GlycA and FEV1 from TWAS

File Name: Supplementary Data 7
Description: Shared Significant Expression-Trait Associations between GlycA and FVC from TWAS

File Name: Supplementary Data 8
Description: Shared Significant Expression-Trait Associations between GlycA and FEV1/FVC ratio from TWAS

File Name: Supplementary Data 9
Description: Shared Significant Expression-Trait Associations between GlycA and PEF from TWAS

File Name: Supplementary Data 10
Description: Shared Significant Expression-Trait Associations between GlycA and asthma from TWAS

File Name: Supplementary Data 11
Description: Shared Significant Expression-Trait Associations between GlycA and COPD from TWAS

File Name: Supplementary Data 12
Description: Shared Significant Expression-Trait Associations between hsCRP and FEV1 from TWAS

File Name: Supplementary Data 13
Description: Shared Significant Expression-Trait Associations between hsCRP and FVC from TWAS

File Name: Supplementary Data 14
Description: Shared Significant Expression-Trait Associations between hsCRP and FEV1/FVC ratio from TWAS

File Name: Supplementary Data 15
Description: Shared Significant Expression-Trait Associations between hsCRP and PEF from TWAS

File Name: Supplementary Data 16
Description: Shared Significant Expression-Trait Associations between hsCRP and Asthma from TWAS

File Name: Supplementary Data 17
Description: Shared Significant Expression-Trait Associations between hsCRP and COPD from TWAS

File Name: Supplementary Data 18
Description: Shared Significant Expression-Trait Associations between Albumin and FEV1 from TWAS

File Name: Supplementary Data 19
Description: Shared Significant Expression-Trait Associations between Albumin and FVC from TWAS

File Name: Supplementary Data 20
Description: Shared Significant Expression-Trait Associations between Albumin and FEV1/FVC ratio from TWAS

File Name: Supplementary Data 21
Description: Shared Significant Expression-Trait Associations between Albumin and PEF from TWAS

File Name: Supplementary Data 22
Description: Shared Significant Expression-Trait Associations between Albumin and Asthma from TWAS

File Name: Supplementary Data 23
Description: Shared Significant Expression-Trait Associations between Albumin and COPD from TWAS

File Name: Supplementary Data 24
Description: Bi-directional instrumental estimates of GlycA, hsCRP, and albumin with lung function parameters, asthma and COPD using MR PRESSO

File Name: Supplementary Data 25
Description: Instrumental estimates of GlycA, hsCRP, and albumin with lung function parameters using MR PRESSO after Steiger filtering
