# Supplementary material for: Genetic association of inflammatory marker GlycA with lung function and respiratory diseases
Source: Nat Commun. 2024 May 4;15:3751. doi: 10.1038/s41467-024-47845-w (PMC11069551; doi:10.1038/s41467-024-47845-w)
Supplement: Supplementary file 5 — Reporting Summary [file 41467_2024_47845_MOESM5_ESM.pdf]

nature portfolio

Corresponding author(s): Yanjun Guo; Weihong Chen

Last updated by author(s): Mar 20, 2024

Reporting Summary

Nature Portfolio wishes to improve the reproducibility of the work that we publish. This form provides structure for consistency and transparency in reporting. For further information on Nature Portfolio policies, see our Editorial Policies and the Editorial Policy Checklist.

Statistics

For all statistical analyses, confirm that the following items are present in the figure legend, table legend, main text, or Methods section.

n/a	Confirmed	
☐	☒	The exact sample size (n) for each experimental group/condition, given as a discrete number and unit of measurement
☐	☒	A statement on whether measurements were taken from distinct samples or whether the same sample was measured repeatedly
☐	☒	The statistical test(s) used AND whether they are one- or two-sided<br>*Only common tests should be described solely by name; describe more complex techniques in the Methods section.*
☒	☐	A description of all covariates tested
☐	☒	A description of any assumptions or corrections, such as tests of normality and adjustment for multiple comparisons
☐	☒	A full description of the statistical parameters including central tendency (e.g. means) or other basic estimates (e.g. regression coefficient) AND variation (e.g. standard deviation) or associated estimates of uncertainty (e.g. confidence intervals)
☐	☒	For null hypothesis testing, the test statistic (e.g. F, t, r) with confidence intervals, effect sizes, degrees of freedom and P value noted<br>*Give P values as exact values whenever suitable.*
☒	☐	For Bayesian analysis, information on the choice of priors and Markov chain Monte Carlo settings
☒	☐	For hierarchical and complex designs, identification of the appropriate level for tests and full reporting of outcomes
☐	☒	Estimates of effect sizes (e.g. Cohen's d, Pearson's r), indicating how they were calculated

Our web collection on statistics for biologists contains articles on many of the points above.

Software and code

Policy information about availability of computer code

Data collection	No software was used for the data collection of this study.
Data analysis	LDSC (https://github.com/bulik/ldsc/wiki/Heritability-and-Genetic-Correlation, Version 1.0.1); , CPASSOC (http://hal.case.edu/zhu-web/), TWAS (http://gusevlab.org/projects/fusion/); GARFIELD (https://www.ebi.ac.uk/birney-srv/GARFIELD/, Version 2); GSMR (https://cnsgenomics.com/software/gcta/#GSMR, Version 1.0.8); TwoSampleMR (https://github.com/MRCIEU/TwoSampleMR, Version 0.5.10)

For manuscripts utilizing custom algorithms or software that are central to the research but not yet described in published literature, software must be made available to editors and reviewers. We strongly encourage code deposition in a community repository (e.g. GitHub). See the Nature Portfolio guidelines for submitting code & software for further information.

Data

Policy information about availability of data

All manuscripts must include a data availability statement. This statement should provide the following information, where applicable:

- Accession codes, unique identifiers, or web links for publicly available datasets
- A description of any restrictions on data availability
- For clinical datasets or third party data, please ensure that the statement adheres to our policy

Data Availability

The summary-level data for GlycA (the UK Biobank [UKB]) used in this study are publicly available at: https://gwas.mrcieu.ac.uk/datasets/met-d-GlycA/; The summary-level data for white blood cell (WBC) used in this study are available at: ftp://ftp.sanger.ac.uk/pub/project/humgen/summary_statistics/UKBB_blood_cell_traits/ and http://www.mhi-humangenetics.org/en/resources. The summary-level data for high sensitivity C-reactive protein levels used in this study are available at: https://www.ebi.ac.uk/gwas/studies/GCST90079026. The summary-level data for fibrinogen used in this study are available at: https://www.ebi.ac.uk/gwas/studies/GCST90019421. The summary-level data for albumin used in this study are available at: https://www.ebi.ac.uk/gwas/studies/GCST90019493. The summary-level data for lung function parameters used in this study are available at: http://ldsc.broadinstitute.org/ldhub/. The summary-level data for asthma and COPD used in this study are available at: https://humandbs.biosciencedbc.jp/en/ with the accession code hum0197, and the GWAS Catalog with the accession code GCST90018795 and GCST90018807, respectively (https://www.ebi.ac.uk/gwas/studies/GCST90018795 and https://www.ebi.ac.uk/gwas/studies/GCST90018807 https://www.ebi.ac.uk/gwas/). The individual level data from the UK Biobank (UKB) are available upon application: https://www.ukbiobank.ac.uk/.

Research involving human participants, their data, or biological material

Policy information about studies with human participants or human data. See also policy information about sex, gender (identity/presentation), and sexual orientation and race, ethnicity and racism.

Reporting on sex and gender	NA. The current study only used the latest large-scale genome-wide summary level data including both sex for glycA, inflammatory biomarkers, lung function parameters, asthma and COPD. Therefore, we did not consider any sex- and gender-based analyses in the present study.
Reporting on race, ethnicity, or other socially relevant groupings	All the participants were from European ancestry and the genotype data were imputed to the 1000 Genomes reference 16. This study uses solely GWAS summary statistics, but all participants who contributed to cohorts provided written informed consent and each of the cohort protocols was approved by a local institutional review board.
Population characteristics	In the present study, we used the latest large-scale genome-wide summary level data for GlycA (N=115,078) from UK Biobank in October 2022. UK Biobank is a deeply phenotyped cohort of 503,325 participants from the 22 study centers across the United Kingdom (UK) during 2006–2010, and all participants were aged between 40 and 69 at recruitment.
Recruitment	UK Biobank is a deeply phenotyped cohort of 503,325 participants from the 22 study centers across the United Kingdom (UK) during 2006–2010, and all participants were aged between 40 and 69 at recruitment.
Ethics oversight	This study uses solely GWAS summary statistics, but all participants who contributed to cohorts provided written informed consent and each of the cohort protocols was approved by a local institutional review board.

Note that full information on the approval of the study protocol must also be provided in the manuscript.

Field-specific reporting

Please select the one below that is the best fit for your research. If you are not sure, read the appropriate sections before making your selection.

☒ Life sciences ☐ Behavioural & social sciences ☐ Ecological, evolutionary & environmental sciences

For a reference copy of the document with all sections, see nature.com/documents/nr-reporting-summary-flat.pdf

Life sciences study design

All studies must disclose on these points even when the disclosure is negative.

Sample size	No sample size calculation was performed. We aimed to include all participants within the UK Biobank, the SpiroMeta Consortium, FinnGen with available summary level data for GlycA, lung function parameters, asthma and COPD.
Data exclusions	No data were excluded from the analyses, except the HLA region was excluded from LDSC analysis.
Replication	Although it is suggested that cross-trait LD Score regression is not biased by sample overlap, we additionally conducted sensitivity analysis using GWAS summary-level data from non-overlapping cohorts (SpiroMeta consortium) to see if the potential sample overlapping biased the genetic correlation estimation between GlycA and lung function. The repeated LDSC using independent data sets showed consistent results with the overall genetic correlation. For example, GlycA showed consistent significant inverse genetic correlation with lung function parameters (FEV1: rg=-0.17, P value=5.84×10-5]; FVC: rg=-0.20, P value=3.08×10-9])
Randomization	This is not relevant to our study since no randomization was performed. We only used summary statistics for GlycA, lung function parameters, asthma and COPD from UK Biobank, the SpiroMeta Consortium, FinnGen.
Blinding	All analyses were derived from de-identified data or summary statistics. The investigators were blinded to group allocation during data collection and analysis.

Reporting for specific materials, systems and methods

We require information from authors about some types of materials, experimental systems and methods used in many studies. Here, indicate whether each material, system or method listed is relevant to your study. If you are not sure if a list item applies to your research, read the appropriate section before selecting a response.

Materials & experimental systems

n/a	Involved in the study
☒	☐ Antibodies
☒	☐ Eukaryotic cell lines
☒	☐ Palaeontology and archaeology
☒	☐ Animals and other organisms
☒	☐ Clinical data
☒	☐ Dual use research of concern
☒	☐ Plants

Methods

n/a	Involved in the study
☒	☐ ChIP-seq
☒	☐ Flow cytometry
☒	☐ MRI-based neuroimaging

Plants

Seed stocks	NA
Novel plant genotypes	NA
Authentication	NA
